# Research on Design and Simulation of Biaxial Tensile-Bending Complex Mechanical Performance Test Apparatus

**DOI:** 10.3390/mi8090286

**Published:** 2017-09-20

**Authors:** Hailian Li, Hongwei Zhao, Chunyang Luo, Lijia Li, He Zhang

**Affiliations:** 1 School of Mechanical Science & Engineering, Jilin University, Changchun 130025, China; hlli12@mails.jlu.edu.cn (H.L.); llj15@mails.jlu.edu.cn (L.L.); hez15@mails.jlu.edu.cn (H.Z.); 2 School of Mechanical Science & Engineering, Beihua University, Jilin 132021, China; luochunyang2004@126.com

**Keywords:** biaxial tensile, bending, combined loading, micro-mechanical properties

## Abstract

In order to realize a micro-mechanic performance test of biaxial tensile-bending-combined loading and solve the problem of incompatibility of test apparatus and observation apparatus, novel biaxial-combined tensile-bending micro-mechanical performance test apparatus was designed. The working principle and major functions of key constituent parts of test apparatus, including the servo drive unit, clamping unit and test system, were introduced. Based on the finite element method, biaxial tensile and tension-bending-combined mechanical performances of the test-piece were studied as guidance to learn the distribution of elastic deformation and plastic deformation of all sites of the test-piece and to better plan test regions. Finally, this test apparatus was used to conduct a biaxial tensile test under different pre-bending loading and a tensile test at different rates; the image of the fracture of the test-piece was acquired by a scanning electron microscope and analyzed. It was indicated that as the pre-bending force rises, the elastic deformation phase would gradually shorten and the slope of the elastic deformation phase curve would slightly rise so that a yield limit would appear ahead of time. Bending speed could exert a positive and beneficial influence on tensile strength but weaken fracture elongation. If bending speed is appropriately raised, more ideal anti-tensile strength could be obtained, but fracture elongation would decline.

## 1. Introduction

Material science, as the leading discipline for all advanced technologies, represents the foundations of the national economy. Nowadays, the micro-mechanical properties of materials are a popular and interesting subject [1,2]. Material failure due to the unclear mechanism of microscopic damage to material is one of the major causes of severe accidents, loss of human life and property damage, and the major reason for the problem is poor material testing capacity. 

In actual conditions, the material is not only subject to a single load. The mechanical properties of the specimen are very different when the same specimen is loaded with different loads. Therefore, the mechanical properties of the materials under multiple loads will provide a more reliable theoretical basis for the design and manufacture of mechanical components.

The whole development process of material in practical conditions from microscopic crack initiation to propagation and macroscopic deformation to fracture may be subject to research in test apparatus. At present, various measuring and test techniques are observed based on AFM (atomic force microscope), SEM (scanning electron microscope), a Raman spectrograph, X-ray diffractometer and metallurgical microscope [3,4,5,6]. In a broad sense, an in situ tensile-bending test is usually used to observe and record the whole process including material crack initiation, propagation and fracture in real time. 

Under normal conditions, such testing is carried out under a high-resolution electron microscope. Such testing as a basic method has laid the foundations for further research on the mechanical performance of material under engineering stress–strain curves. Accordingly, Lawrence Berkeley National Laboratory (LBNL) and Lawrence Livermore National Laboratory (LLNL) [7] carried out a series of related studies on specimens including nanoscale tube, nanowire and other objects of extremely tiny size under uniaxial tensile mode by focused ion beam (FIB) technology. In addition, Sanchez [8] set up an elastoplastic mathematical model with plane strain flow for metallic material under tensile load and cyclic bending load and analyzed the steady flow change pattern of stress, strain, strain rate, geometric flow change, resilience and residual stress of metallic material in the plane strain state. In view of the biaxial tensile test of metal materials, Ma and his colleagues conducted extensive research [9]. The micro-indentation technique was applied to prepare the initial indentations as embedded surface defects at the gauge length section and central section of a cross-shaped AZ31B magnesium alloy specimen. Via the observation by an optical microscope with three-dimensional imaging and measurement functions, the changing laws of the indentation topographies under uniaxial and biaxial tensile loads were discussed. Kubo et al. [10] recently developed a new biaxial tensile testing system, combining SEM, which could be used in observation. These studies were conducted based on micro electro mechanical system (MEMS) technology [11].

At present, in situ tensile tests on bulk material are carried out by means of imaging and the characterization function of X-ray diffraction (XRD), SEM and AFM [12,13,14], and a sensor is utilized to carry out real-time monitoring. Since charge-coupled-device (CCD) is a key carrier for optical imaging information of the specimen surface, and compared with SEM, it is still able to carry out high magnification imaging at longer working distance, it is often applied as an auxiliary apparatus in forming test apparatus. In the field of research on bulk material, Bale et al. [15] and others, using an X-ray diffractometer, successfully observed the silicon carbide polymer at high tensile temperatures of more than 1600 °C, to overcome the ultra-high temperature in situ observation problems. To study the tensile test under CCD, Fethi Abbassi [16] conducted uniaxial tensile and biaxial tensile tests on uniaxial tensile specimens and cross-shaped specimens using a biaxial tensile tester for tensile test studies under CCDs. The industrial CCD three-dimensional image method included analysis of the failure of the specimen and the micro-hole on the specimen in a two-way extension of the process.

Thus, the in situ test apparatus technology of a single load has evolved, but in practice the material and its products are usually subject to complex load [17]. The in situ test apparatus of a single load can only accurately measure the mechanical properties of the material under a single load, and it is difficult to accurately describe the relationship between the stress and strain of the specimen under the composite load. Therefore, research on in situ test technology under the composite load, as well as the development of the complex load environment which can be measured under the mechanical properties of the test apparatus, has become the focus of attention at home and abroad.

With regard to the above-mentioned problems, this paper presents a design proposal on modular test apparatus compatible with SEM or CCD for in situ tensile-bending-combined load at nanoscale, and introduces every component and function of the test apparatus. Deformation of the specimen and strain distribution in two-way tensile and tensile-bending-combined conditions was analyzed by finite element simulation to obtain a range of reasonable structure sizes. In this design proposal, analysis and processing of the specimen size and test data were properly studied to ensure accuracy of the test result. Thus, problems such as scattered test results that exist in in situ tensile-bending test apparatus, as well as limitations in the single load test on bulk material at and above millimeter scale, could be solved. 

## 2. Description of Research and Development of Test Apparatus 

**Figure 1:**
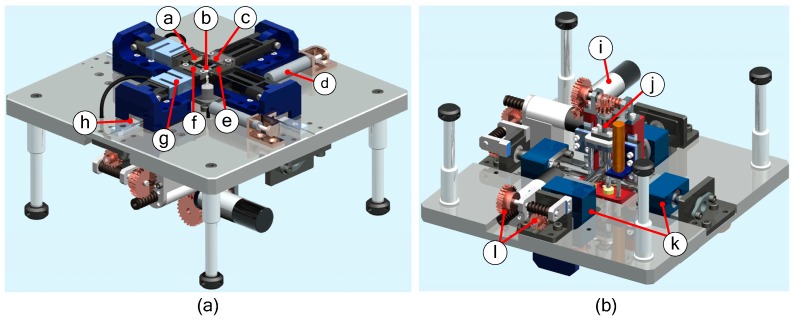
Mechanical properties of the biaxial tensile-bending composite test apparatus. (**a**) The top of the apparatus structure; (**b**) The bottom of the apparatus structure. ⓐ Clamp D; ⓑ Specimen; ⓒ Clamp A; ⓓ Displacement sensor; ⓔ Clamp C; ⓕ Clamp B; ⓖ Force sensor; ⓗ Guide rail; ⓘ Servo motor; ⓙ Bending ball screw; ⓚ Two-way ball screw; ⓛ Worm gear reducer.

In this paper, a novel in situ test apparatus was designed with the following functions: uniaxial pure tension test; air bending experiment; biaxial tension experiment; uniaxial tension-bending-combined loading experiment; biaxial tension-bending-combined loading test. The structure of this apparatus is shown in Figure 1. The test apparatus designed in this paper is different from the existing single-load test apparatus. It can realize a single load or a combined load on the same apparatus and can make the data more persuasive and closer to the actual load in the actual working conditions. The test apparatus is primarily composed of three major components, including a servo drive unit, clamp unit and test system. The servo drive unit contains tensile drive and bend drive. In tensile drive, the tensile base is driven by a turbo-worm reducer and left/right-hand ball screw to produce a tensile effect. The bend drive principle is identical to the tensile drive principle. The test system is based on the SEM imaging device and control system, adopting the STM32 (STMicroelectronics, Geneva, Switzerland) embedded system for control. In addition, auxiliary parts, including the base and jack, are the primary supporting components for the test apparatus. This testing apparatus with compact structure, small size and multiple functions could be compatible with SEM observation. Since this apparatus could realize quasi-static loading [18] (2.4 μm/s loading), the focus-out problem of the observation device in dynamic observation could be effectively solved. Thus, the inner deformation of the test-piece could be dynamically observed in real time, and the mechanical performance of the material could be analyzed more accurately.

### 2.1. Drive Unit 

In the existing complex loading mode, the specimen is subject to loading by different axes or tension at different heights, in order for the complex load, such as tensile and bending combination or compression and shearing combination, to appear inside the material to meet the conditions of the complex load test. The apparatus is based on a novel idea rather than research on complex load based on existing technology. The drive system has an X, Y biaxial tensile structure and a Z axial bend structure in the design to generate horizontal tension along the X and Y directions, as well as vertical bend force along the Z direction, respectively, so as to perform uniaxial and biaxial testing, three-point bending testing and biaxial tensile-bending-combined loading testing.

The drive unit adopts a maxon dc servo motor with low speed, large torque and high precision, and the reducer is composed of two-stage 30:1 worm gear so that the total deceleration rate reaches 900 and quasi-static loading at 2.4 μm/s low load speed is realized. In this loading mode, the ‘quasi-static’ loading method with a big deceleration ratio was used to effectively solve the focus-out problem in the measuring instrument part of the testing machine caused by the change of distance between the surface of the test-piece and CCD during the bending experiment. In order to ensure that the specimen center is fixed and motionless in the biaxial tensile test, the test apparatus adopts a two-way ball screw to carry out one-way rotation to drive the specimen clamp to move at the same speed and in different directions along the guide rail. The linear guide rail and sliding block are assembled in the cross groove on the base plane and work with the ball screw to achieve transmission. Meanwhile, the bilateral ball screw with a self-locking function was adopted so as to realize dynamic and static observation modes in the experiment. Based on this drive mode, the developed apparatus is able to load and unload tension by switching the rotational direction of the servo motor through pulse and direction control in a convenient and comfortable way.

### 2.2. Specimen Clamp Unit 

**Figure 2:**
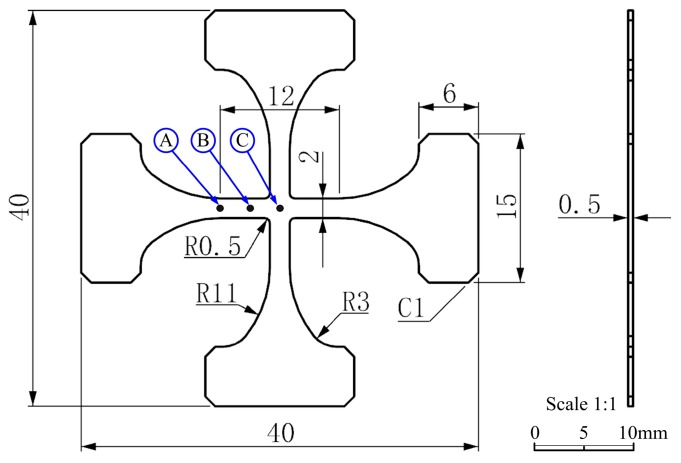
Specimen structure size.

Clamping apparatus could simply and precisely clamp the specimen. According to the People’s Republic of China Apparatusry Industry Standard (JB/T13223-2017) [19], the rectangular section was selected in the specimen of this test apparatus. The specimen of the rectangular section is beneficial for reducing the macro stress concentration so that premature failure and invalidness of the specimen in testing could be avoided. In general, the uniaxial sheet-type tensile specimen is H-shaped. Both wide ends are used as the clamping part of specimen, and the narrow middle part has parallel length. Big arc transition is set between the two parts to reduce the stress concentration of the fixed end of the specimen. In this paper, the test apparatus designed was biaxial lateral tension, which was like simultaneously bending two H-shaped specimens. Hence, the specimen was designed as a vertical arrangement of two H-shaped specimens; in other words, a cross-shaped specimen. Limited by the size of the test chamber of Zeiss-EVO18 SEM (Carl Zeiss Microscopy, Peabody, MA, USA)and the structure of the test apparatus, the size of the specimen was designed as 40 mm × 40 mm × 0.5 mm. Mark width and mark length were 2 and 12 mm, respectively, as shown in Figure 2. In the experimental specimen, A, B and C were defined, respectively as the characteristic points of the simulation analysis. There may be stress concentration in the intersection of the specimen, including the marking section and loading section, so the position is defined as the characteristic point A. The center of the cross specimen is the point of action of the bending load, defined as the characteristic point C. Between the feature points A and C is the smallest cross-sectional area of the specimen. The middle position of this region is set as the feature point B. 

**Figure 3:**
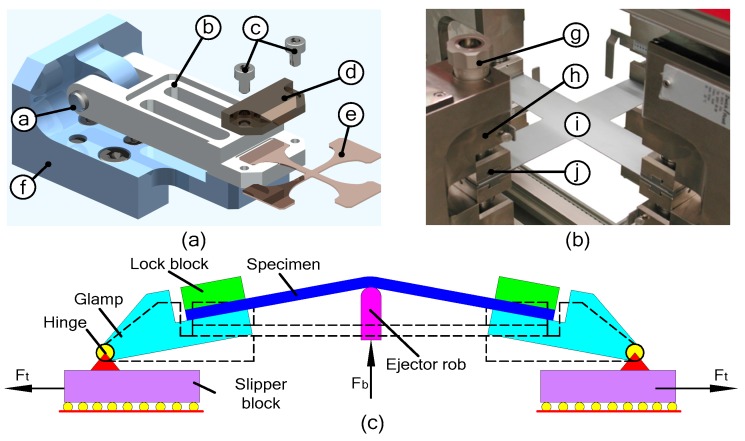
Clamping program for the specimen. (**a**) Schematic diagram for specimen clamping; (**b**) Loaded schematic of the clamping unit bender; (**c**) Clamping form of the existing commercial test apparatus. ⓐ Hinge; ⓑ Clamp; ⓒ Compression screw; ⓓ Lock block; ⓔ Specimen; ⓕ Slipper block; ⓖ Compression screw; ⓗ Clamp; ⓘ Specimen; ⓙ Lock block.

The clamping device was composed of a sliding base, hinge, clamp, lock block and compression screw, as shown in Figure 3a. The clamp was hinged with a sliding base. When the specimen was applied by bending load F_b_, the fixture body would turn around along the hinge in the process of specimen bending. The fixture body and clamping position of the specimen was always tangent to avoid the extra bending deformation of the end face of the fixture. Thus, the bending position would only emerge in the action point of the top elbow of the specimen, as shown in Figure 3c. On the clamp, the positioning groove was processed according to the outline of the clamping position of the specimen. The specimen was put into the groove so that its positioning accuracy could be guaranteed. A compression screw was used to fasten the lock block and clamp. Thus, the specimen was precisely fixed to the clamping apparatus. Depending on the lock block, the specimen in the groove of the clamp could fit the clamp. Adequate tension force was provided by the contact between the closing position of the T-shaped groove and the specimen. Figure 3b shows the clamping scheme for the cross-shaped test-piece in the existing commercial testing apparatus. The specimen is fixed by the fiction provided by positive pressure. The accuracy of positioning and the reliability of clamping are considered. The clamping device could not rotate. Thus, extra bending deformation would emerge, and the resulting measurement would be influenced. In addition, in the design of a unique clamping device in this test apparatus, the characteristics of the hinge were flexibly used to realize a pure three-point bending experiment. Meanwhile, the bending experiment could also be accurately simulated, and the authenticity of the acquired data in the experiment would be improved.

### 2.3. Test System 

Different from current single loading, this test apparatus could bear both single loading and combined loading. Thus, the data obtained would be more convincing and closer to the real loading that the work-piece bears. The control core for the test apparatus is the STM32 embedded system. Its chip has high-speed calculation capability and information processing capability so that the flexibility, reliability and real-time performance of the apparatus are greatly improved. A PM11-R1-10L (XiYu Co., Ltd., ShangHai, China)ultra-high precision linear transducer is adopted in the displacement transducer to meet the requirement of precision. It is characterized by a high integration level, small volume and favorable stability with optional multi-stage precisions. Its highest precision is 0.001 mm, and repeatability precision is 0.01%. It has three types of standard output signals, including resistance, current and voltage. Among them, the voltage range is 0–5 V and 0–10 V, and the current range is 0–20 mA and 4–20 mA. 

The JLBS-V (JiNuo Co., Ltd., Bengbu, China) pressure transducer is adopted on the X axis and Y axis of the test apparatus. Its outline is “S-shaped” to achieve switching between tensile load measurement and compressive load measurement. The transducer adopts a foil gage against the alloy steel elastomer, characterized by high measuring precision, favorable stability, small temperature drift, good output symmetry and compact structure. The Z axis adopts the SM609-A (Sensehr technology Co., Ltd., Shenzhen, China) pressure transducer made of stainless steel, with a IP65 level of protection, having been widely used in tension and compression operation, such as a push–pull dynamometer, hopper scale and industrial measuring system. 

**Figure 4:**
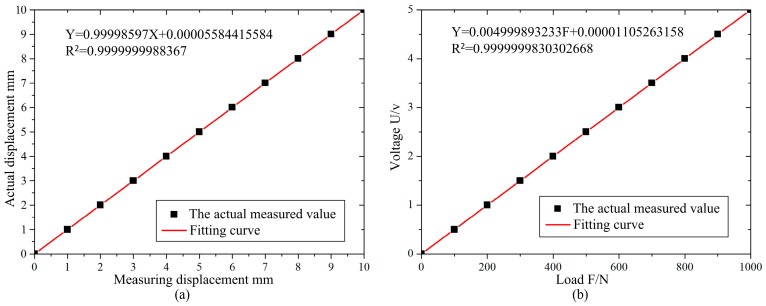
Sensor calibration curve diagram. (**a**) Displacement sensor calibration; (**b**) Force sensor calibration.

The linear displacement sensor and the force sensor in the test apparatus are calibrated using the laser displacement sensor and the standard weight. Figure 4 shows the calibration curve of the displacement sensor and the force sensor. The linear correlation coefficient of the sensor is 0.99999, which can meet the test requirement of the test apparatus.

Since the test apparatus is characterized by a compact structure, small volume and automatic operation, it may be placed under a microscope to observe the change process of the metallic crystal structure when material is loaded. In other similar functional equipment, it is only possible to place material under the microscope to observe static change of the metallic crystal structure after material loading is over.

**Figure 5:**
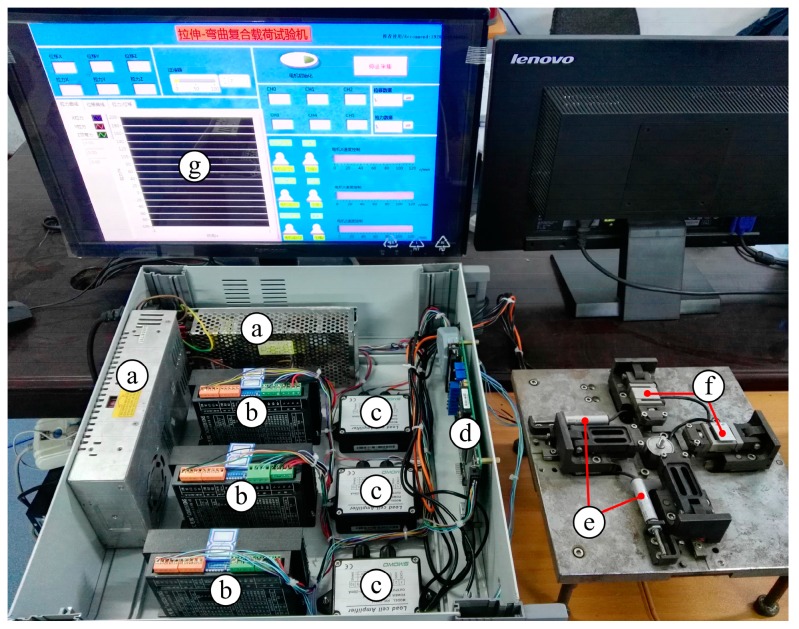
Loading test system diagram. ⓐ Power supply; ⓑ Motor control; ⓒ Signal amplifier; ⓓ Data acquisition card; ⓔ Displacement transducer; ⓕ Force transducer; ⓖ Upper computer.

During operation, after relevant parameters are set by the upper computer, information will be transmitted to the lower computer by a signal acquisition card (STM32 embedded system). The lower computer will convert the digital signal received to analog signal and then transmit the signal to the motor driver to drive the motor to operate. In addition, the displacement transducer and tension/pressure transducer will feedback the acquired signal to the lower computer on a real-time basis so as to adjust the operating parameters of the motor after the PID control algorithm process, including rotary speed and torque, to make the system a closed loop. In addition, the lower computer will also transmit the signal received to the upper computer on a real-time basis so as to record and calculate various parameters and draw curves. By controlling motor operation, single load or complex load could be achieved (tensile function on the X axis or Y axis, namely uniaxial tension; or tensile function on the X axis and Y axis, namely biaxial tension, bending function on the Z axis, uniaxial tensile and bending combination, biaxial tensile and bending combination). Figure 5 shows the loading test system diagram.

### 2.4. Test Condition for Test Apparatus 

It is observed from the design principle and structure design form that this test apparatus has certain test conditions. Its design size as well as shape and size of the specimen to be tested will match with the design principle and characteristics of the test apparatus.

In order to make it convenient for the test apparatus to load in the test process and achieve analysis in signal load mode as well as carry out separate loadings or successive loadings in two or more load modes and offer effective test data on the mechanical performance and deformation and the damage mechanism for material under combined load, the design adopts a drive mode based on loading by staggered tension on a horizontal cross along the X axis and Y axis and a drive mode based on loading by vertical bending along the Z axis with reduced load analysis. Accordingly, requirements are proposed for the design shape of the specimen to be tested. The test apparatus requires the cross specimen to be tested, with the fillet prepared on the cross joint to prevent inaccuracy of the test data due to stress concentration at the joint of the specimen in the test process. 

The apparatus needs to work with a scanning electron microscope. However, the electron microscope to be adopted will have strict requirements regarding the overall dimension of the test apparatus due to its special observation environment. Since the chamber of the electron microscope is 320 mm in diameter, the entire design dimension is 240 mm × 240 mm × 140 mm. Thus, it is ensured that the test apparatus is able to work with the electron microscope in the test process in an effective way, and a proper test environment is guaranteed for data testing. 

On the other hand, the specimen of the apparatus was photographed by the Nikon SMZ745 (Nikon Instruments, Tokyo, Japan) optical microscope during the tensile process. The specimen is subjected to testing by calibration of the observation height from lens to specimen to obtain the preferred observation area for specimen deformation and to determine the preferred dimension of the specimen to be tested by the test apparatus. The design proposal presents an analog simulation analysis of the theoretical deformation of the specimen to determine the design dimension of the specimen according to data analysis. 

## 3. Test Apparatus Simulation Analysis 

**Figure 6:**
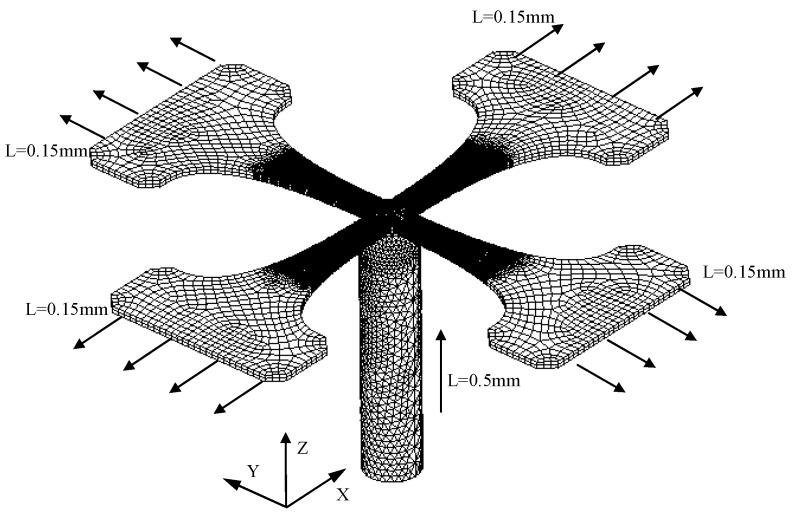
Finite element simulation model.

In order to further understand the elastic deformation and plastic deformation distribution of the test specimen, the test area is better planned to locate the effective test area and improve the test precision of the test apparatus. In this paper, the simulation test is carried out. According to the test process, the finite element model is established by using finite element analysis software ANSYS (ANSYS 10.0, ANSYS, Inc., Canonsburg, PA, USA). As shown in Figure 6, considering the nonlinear characteristics of aluminum alloy material, the mechanical properties of the test material (as shown in Table 1) are input into ANSYS to simulate the material properties [20]. The specimen structure is consistent with the real specimen; the specimen is subjected to the biaxial tensile load of X and Y, respectively. The bending load is applied in the Z direction by the top elbow at the bottom, and the bending effect.

### 3.1. Biaxial Tensile Mechanical Performance Analysis 

**Figure 7:**
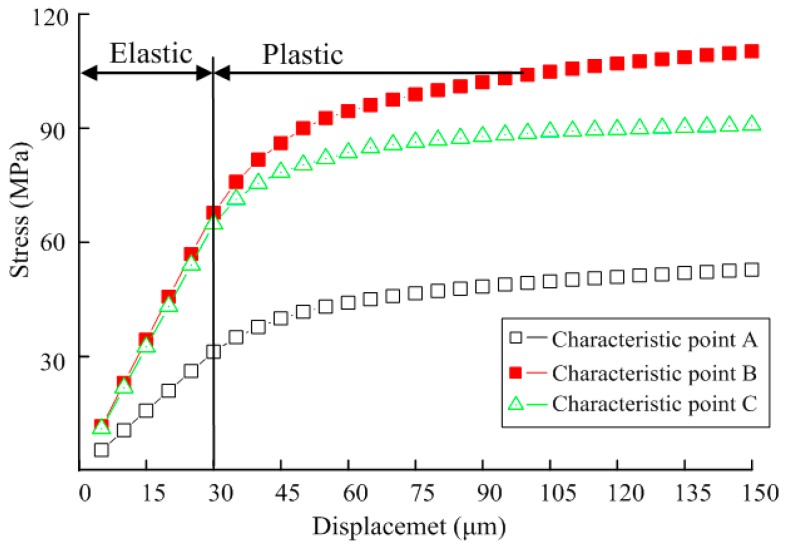
Displacement dependent variation for stress of the specimen.

During biaxial tension, the displacement-dependent stress variation curve at characteristic points A, B and C on the specimen were as shown in Figure 7. It is observed that the stresses on three characteristic points in the elastic stage show linear variation. In the case that displacement on both sides δ < 30 μm and that the specimen is in the linear elastic stage, tensile stress and nodal displacement show linear variation, with the variation rate of stress at B and C larger than that at A. Accordingly, when the test is carried out within the elastic range, it is better to take B and C as test points. When displacement δ > 30 μm on both sides，the test mock-up is in the plastic stage, and tensile stress and nodal displacement show non-linear variation. 

**Figure 8:**
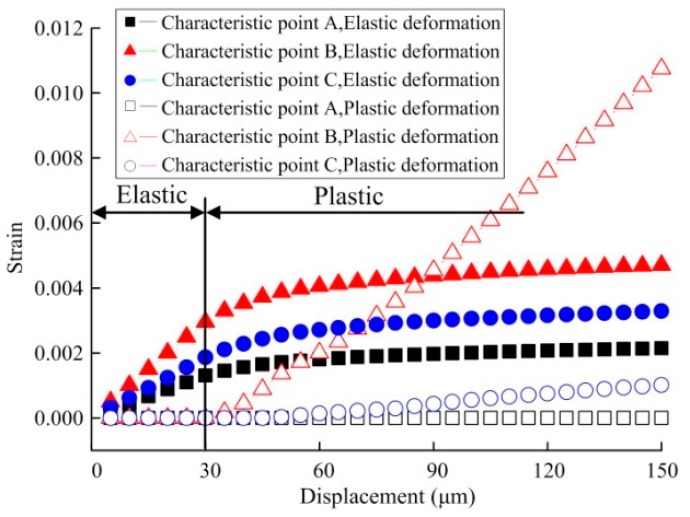
Displacement-dependent variation for strain of the specimen under biaxial tensile load.

Displacement-dependent variation curves for elastic strain and plastic strain at characteristic points A, B, C were shown in Figure 8. It is observed that since structural stress at A is small with a maximum of only 58 Mpa, far from its elastic limit, elastic strain only occurs at A positional structure without plastic strain. At B, in the linear elastic stage, its elastic strain shows linear increase with nodal displacement, and the plastic strain in the stage is 0 all the time. When displacement δ > 30 μm on both sides, the increased rate of elastic strain gradually decreases, the elastic strain curve gradually becomes smooth, and plastic strain rapidly increases. Plastic strain at C also shows an increasing trend, with a variation rate far smaller than that at B. Therefore, it is considered that plastic deformation would firstly take place at B when testing plastic deformation.

### 3.2. Tensile-Bending-Combined Mechanical Performance Analysis 

**Figure 9:**
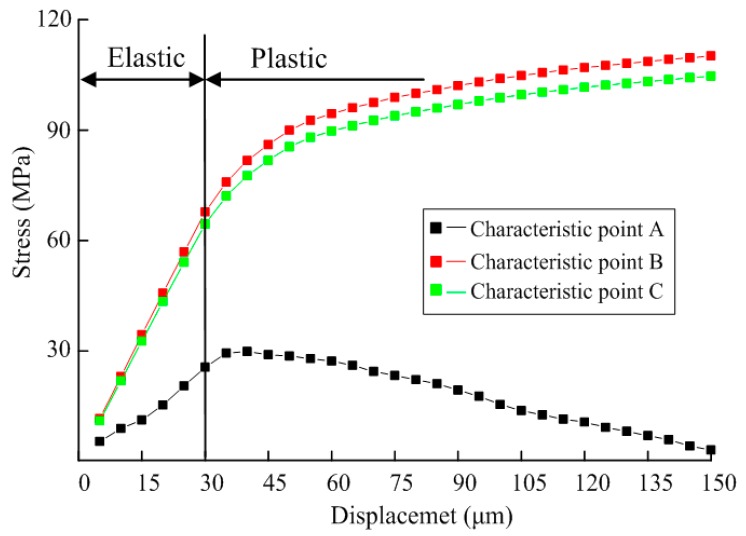
Displacement-dependent variation for stress of the specimen.

Under the biaxial tensile-bending-combined effect, the displacement-dependent variation curves for stress at characteristic points A, B, C are shown in Figure 9. It is observed that the stress at three characteristic points in the elastic stage shows linear variation. In the case that displacement on both sides δ < 30 μm and that the specimen is in the linear elastic stage, tensile stress and nodal displacement show linear variation. Since the specimen deformation is small, the bend is not obvious in the middle. Although stress at C slightly increases compared with that in the tensile state, the stress variation rate at B is still higher than that at C, with minimum variation rate appearing at A. In the case that displacement on both sides δ > 30 μm, some structures of the specimen are in the plastic stage, and tensile stress and nodal displacement show nonlinear variation. 

The specimen is simultaneously subjected to tension and bending at A. In addition, bending at A generates compressive stress. Therefore, as bending deformation increases, stress gradually decreases. B mainly bears pure tension. C bears both tension and bending with stress superposition. Accordingly, at C, stress obviously increases compared with that under pure tension, and the stress variation rate also gradually increases as bending deformation increases.

**Figure 10:**
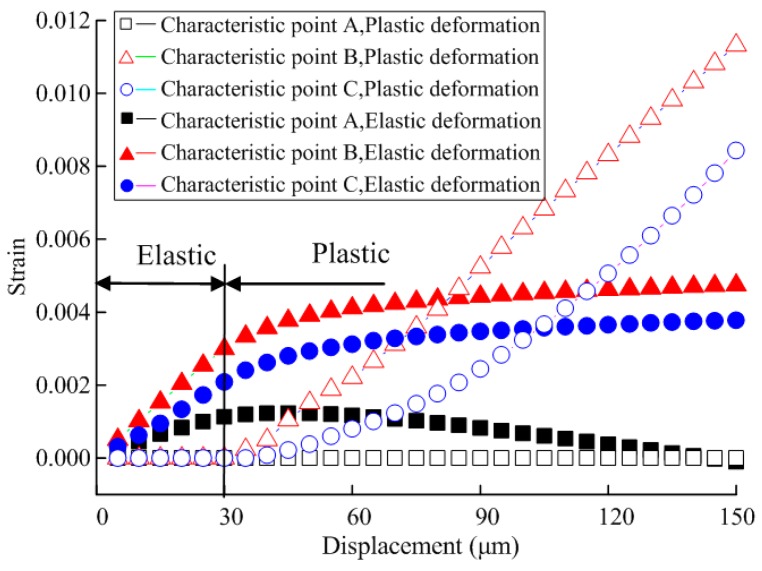
Displacement-dependent variation for strain of the specimen under combined load.

Displacement-dependent variation curves for elastic strain and plastic strain at characteristic points A, B and C are shown in Figure 10. It is observed that compared with pure tension, in the linear elastic stage of the specimen, the elastic strain variation rate at C increases, while the elastic strain variation rate at A decreases, influenced by bending. 

Since structural stress at A is small with a maximum of only 30 Mpa, far from its elastic limit, the structure at A only generates elastic strain, without plastic strain. At B, under little influence of bending, its variation pattern is identical to that under the effect of pure tension. Elastic strain shows linear increase with nodal displacement, and plastic strain in the stage is 0 all the time. In the case that displacement on both sides δ > 30 μm, the elastic strain increase rate gradually decreases, and the elastic strain curve gradually becomes smooth, but plastic strain rapidly increases. In the plastic stage, since C is obviously influenced by bending, plastic variation at this point rapidly increases. 

### 3.3. Distribution of Stress on the Specimen for the Test Apparatus 

**Figure 11:**
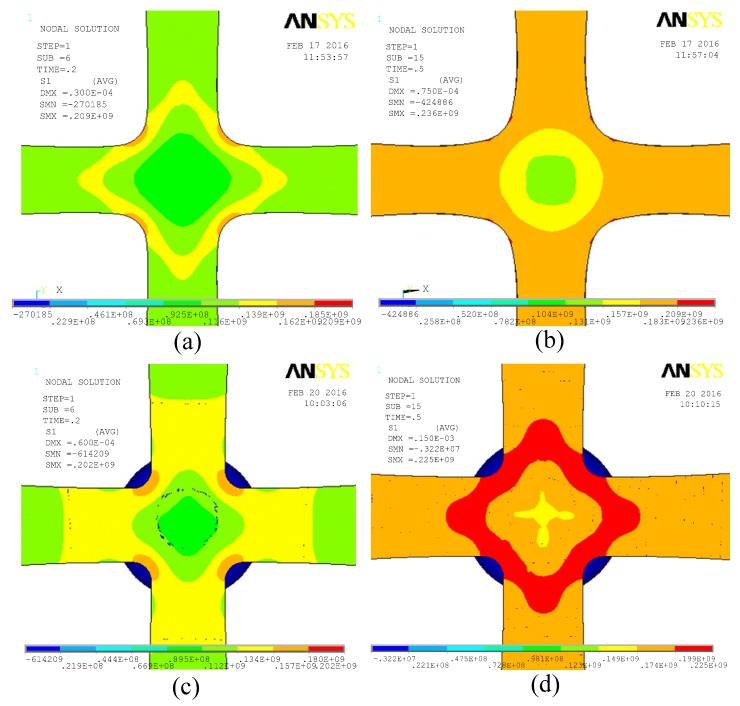
Test apparatus prototype of the first principal stress cloud. (**a**,**b**) the first principal stress of the structure under the pure tensile condition; (**c**,**d**) the first principal stress of the structure under the tensile-bending composite condition. (**a**) δ = 30 μm; (**b**) δ = 75 μm; (**c**) δ = 30 μm; (**d**) δ = 75 μm.

In order to better understand the distribution of stress on the specimen for the test apparatus, the distribution of structural stress on the specimen in the pure tensile condition as well as the tensile-bending-combined condition is subjected to calculation, respectively, as shown in Figure 11.

Given the pure tensile condition, first primary stress shows rhombic distribution in the center of the specimen. As tensile displacement increases, the rhombic area gradually spreads. Stress is relatively small in the center of the specimen and concentrates in the rhombic stress area and cross joint of the specimen. In the case that δ = 30 μm, the test specimen just reaches the elastic limit, and an obvious stress concentration area appears in the cross joint of the specimen. In the case that δ = 75 μm, the test specimen reaches the plastic deformation stage, stress unobviously increases in the middle of the specimen, and a small stress concentration area appears in the cross joint of the specimen. 

Given the tensile-bending-combined condition, the distribution of first primary stress of the specimen is similar to the distribution under the pure tensile condition, shown as a diamond-shaped distribution. The stress is relatively small at the central position of the specimen, while a lot of stress concentrates in the diamond annular region. Therefore, given these test conditions, it is believed that as testing loading increases, plastic deformation or fracture should firstly appear in the structure of the region where stress concentrates. In testing, this region should be the focus when the emergence and extension tendency of a structure crack is observed.

## 4. Experimental Research of Test Apparatus

In order to verify the reliability of the test apparatus, the content of this section adopts the comparison method with the commercial apparatus. The commercial apparatus comparison test and the repeatability test are carried out respectively. The relative deviation of all the parameters is less than 5%. This test apparatus has a high test accuracy and stability.

### 4.1. Biaxial Tension Test under Different Pre-Bending Loading

The specimen made by 1 mm thick 6061-O aluminum alloy material was selected to conduct four groups of biaxial tension test under different pre-bending loading. Both X and Y axial tensile speed is 2.5 μm/s. In order to guarantee the reliability of pre-bending force loading, in four groups of tests, after the specimen was reliably clamped, 20 N pre-tension loading was applied along the X axis and Y axis. Based on this, bending loading was applied. Bending loading was stopped when it reached a default value. Then, biaxial tension loading started till the specimen fractured. The loading parameters of four groups of tests are shown in Table 2.

**Figure 12:**
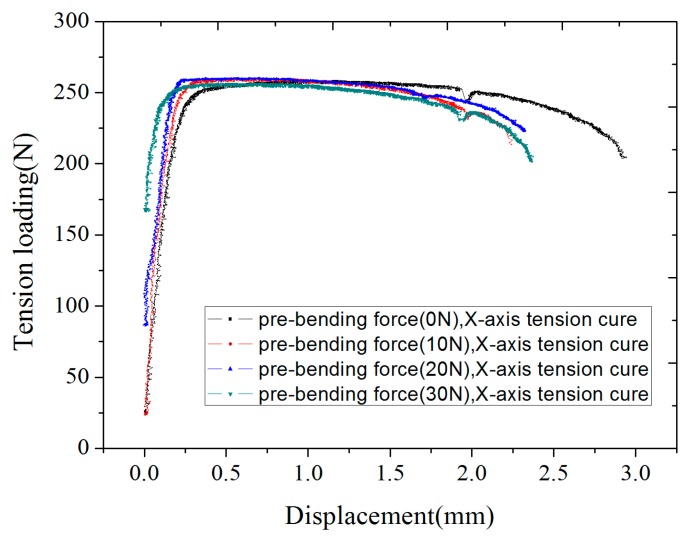
X-axis tension curves under different bending loading.

X-axis tension curves under different bending loading in four groups were drawn together for analysis, as shown in Figure 12. According to the curves, since 0, 10, 20 and 30 N pre-bending forces were loaded, respectively, different changes of tension curves appeared.

Based on the test results and the analysis on the curves, the following conclusions can be drawn:

(1) As the pre-bending force increases, the elastic deformation phase of the curve becomes shorter. When the pre-bending force is 0 N, the elastic deformation work is used entirely for elastic deformation, so the path of the elastic deformation phase is long. For the same specimen, when a certain amount of bending force is applied, elastic deformation work can be regarded as two parts: bending deformation and elastic deformation. Bend generates bending deformation. In formal tension, additional loads are needed to overcome this part of the bending deformation so that this bending part could be straightened and then made tense again. The bigger the pre-bending force, the bigger the bending degree of the specimen, the longer the bending correcting process, the greater the force for overcoming bending deformation, the less the loading used for elastic deformation, and the shorter the phase of tension elastic deformation.
(2) As the pre-bending force increases, the force to start generating elastic deformation increases, and the slope of the elastic deformation curve increases slightly. According to Hooke’s law *σ* = *Eε*, the elastic modulus of the material increases slightly. As pre-bending force increases, the force required to overcome bending deformation increases, and the force needed for elastic deformation increases. In other words, the stiffness of materials increases, elastic deformation becomes difficult, and elastic modulus value increases.
(3) As the pre-bending force increases, the anti-tensile strength of the material would not obviously change, but the position on the curve changes. When the pre-bending force is 0 N, the anti-tensile strength occurs in the mid-stage of the plastic deformation. When the pre-bending force is 10 N, 20 N and 30 N, the anti-tensile strength is shifted to the left, which basically occurs in the initial phase of the plastic deformation. The bigger the pre-bending force, the more obvious the phenomenon. For the plastic material of 6061Al without an apparent yield stage, the stress when plastic strain is 0.2% is usually used as the yield limit stress [22]. After pre-bending force is loaded, the ability of material to resist elastic deformation increases. Compared with no pre-bending force, elastic deformation needs larger load so that the yield limit emerges in advance.

The fracture position of the specimen is near the characteristic point B shown in Figure 2, which is consistent with the simulation analysis. The specific mechanical parameters obtained from the test are shown in Table 3. By comparing the standard values, it can be seen that all the errors are less than 5%, and the test data are in good agreement with the mechanical properties of the 6061-O aluminum alloy. The test data are accurate and reliable.

**Figure 13:**
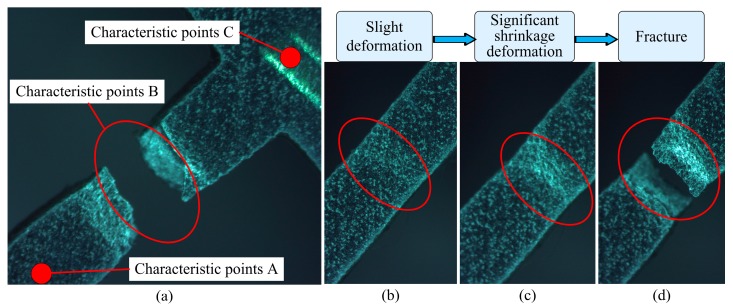
Fracture specimen image. (**a**) The state of the fracture specimen; (**b**) Slight deformation; (**c**) Significant shrinkage deformation; (**d**) Fracture.

**Figure 14:**
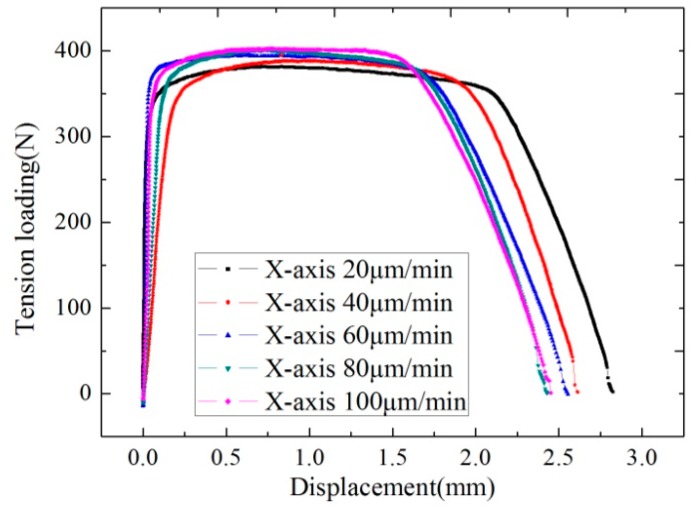
Comparison of loading displacement curves under different biaxial tension speeds (X-axis).

The specimen was photographed by the Nikon SMZ745 optical microscope during the stretching process. It can be seen from Figure 13a that the specimen fracture occurred at the position where the feature point B was located. This result is consistent with the simulation analysis. Figure 14b–d show the process of the fracture.

### 4.2. Biaxial Tension Test under Different Speeds

On a 0.5-mm thick hot-rolled TA1 titanium alloy plate, the material tensile strength of 407 Mpa [23] was selected as the specimen to test the mechanical performance of biaxial tension under different speeds. The tension speed was 20, 40, 60, 80 and 100 μm/min in ascending order. The initial parameters and related records of biaxial tension were shown in Table 4.

According to experimental data, X and Y axes have consistent curve change rules. Due to the rolling direction of material along the X-axis, material would fracture along the Y-axis ahead of time. Curve fluctuation would not appear due to the fraction in another axis. Hence, the tension curves corresponding to the X-axis were drawn together for analysis, as shown in Figure 10. As tension speed rises, the anti-tensile strength of the material would increase, from 381.80 Mpa in 20 μm/min to 402.53 Mpa in 100 μm/min. Meanwhile, as tension speed rises, the fracture phase of the curve would be gradually ahead of time. Therefore, tension speed would exert a positive influence on anti-tensile strength and could weaken the fracture extension rate. If tension speed is appropriately raised, more ideal anti-tensile strength would be obtained, but the fracture extension rate would decline [24].

**Figure 15:**
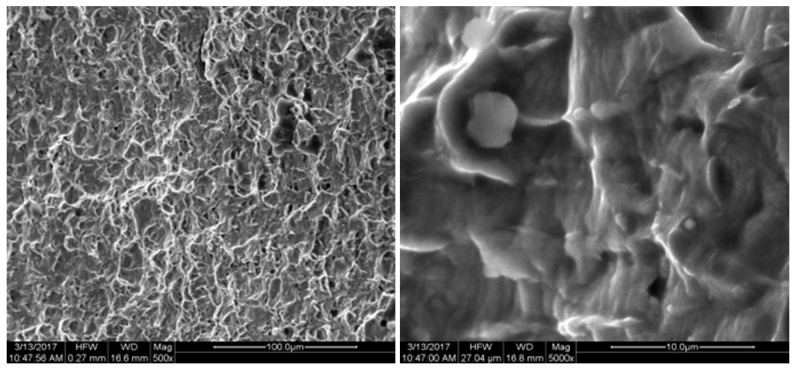
X-axis fracture under the tension speed of 20 μm/min.

**Figure 16:**
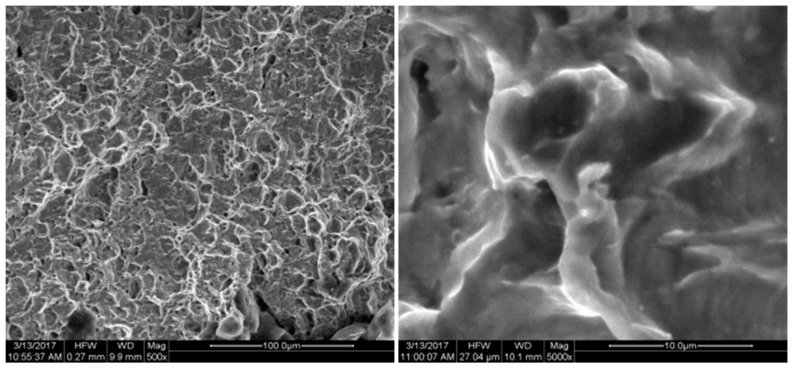
X-axis fracture under the tension speed of 40 μm/min.

**Figure 17:**
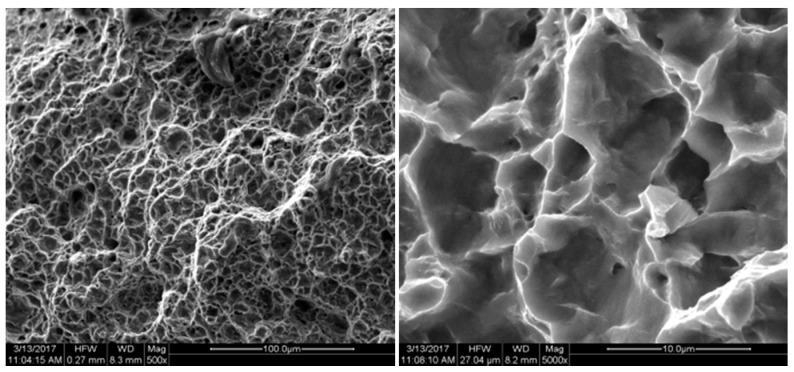
X-axis fracture under the tension speed of 60 μm/min.

**Figure 18:**
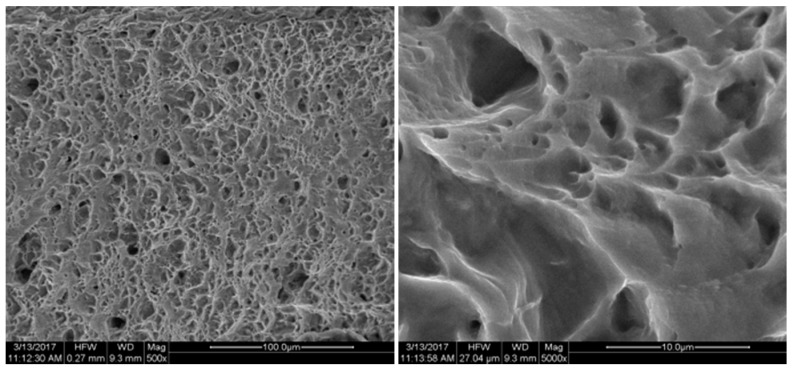
X-axis fracture under the tension speed of 80 μm/min.

**Figure 19:**
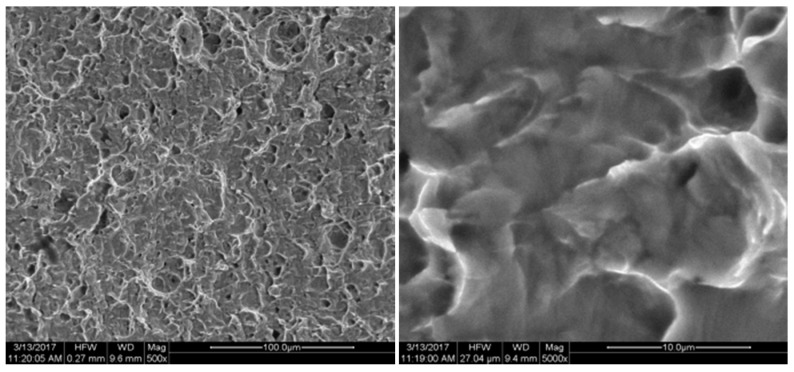
X-axis fracture under the tension speed of 100 μm/min.

The fracture position and the biaxial tensile fracture position of the specimen are observed at the characteristic point B position shown in Figure 2, which is consistent with the conclusion of the simulation analysis. FEI Quanta 200 SEM (FEI Technologies Inc., Hillsboro, OR, USA) was used to conduct fracture analysis for the cracked specimen. The fracture was shown in Figure 15, Figure 16, Figure 17, Figure 18 and Figure 19. Under different tension speeds, the fracture of the titanium alloy specimen had plenty of dimples and tear edges [25,26]. The characteristics of the ductile fracture were shown. However, there was also apparent difference. As tension speed rose, the size of the dimple would reduce. It indicated that the plasticity of material would reduce with the increasing of tension speed. The dimple was biggest when tension speed was 10 μm/min while it was smallest when tension speed was 90 μm/min. The sizes of the dimples under other speeds were between them. This indicated that the material had optimum plasticity at minimum tension speed. The results of the fracture analysis were consistent with those of the tensile curves.

## 5. Conclusions

In this paper, a kind of ‘quasi-static’ loading biaxial tensile-bending-combined micro-mechanical performance test apparatus was proposed. Firstly, the working principle and major functions of key constituent parts of the test apparatus, including the servo drive unit, clamping unit and testing system, were introduced. Then, finite element simulation analysis was conducted for the mechanical performance of the cross-shaped specimen under bilateral tension and tensile-bending-combined loading. According to the analysis, the distribution of elastic and plastic deformation of different positions on the specimen was obtained, and valid testing regions were determined. It was beneficial for improving the testing accuracy of the test apparatus. Finally, this apparatus was used to carry out tension testing under different pre-bending loading and different speeds. The images of the fracture of the specimen under SEM were acquired and analyzed. It was indicated that as pre-bending force increases, the elastic deformation phase would gradually shorten, the slope of the elastic deformation curve would slightly increase, and the yield limit would appear ahead of time. Tension speed could exert a positive influence on anti-tensile strength but could weaken the fracture extension rate. If tension speed is appropriately raised, more ideal tensile strength would be obtained, but the fracture extension rate would decline. With practicability and enlightenment, this test apparatus could be used as a reference for micro-mechanical performance testing under combined loading.

## Figures and Tables

**Table 1:** Mechanical properties of the test specimen material [21].

Material	ElasticModulus (MPa)	TensileStrength (MPa)	YieldStrength (MPa)	Poisson’s Ratio
6061-O	68.9	124	55.2	0.33

**Table 2:** The parameters in the biaxial tension test under different pre-bending loading.

No.	Pre-Tension Force (N)			Pre-Bending Force (N)
	X	Y	Z	Z
1	20	20	0	0
2	20	20	0	10
3	20	20	0	20
4	20	20	0	30

**Table 3:** Comparison of Biaxial Tensile Test Parameters.

Compare the Project	Elastic Modulus (GPa)	Tensile Strength (MPa)	Yield Strength (MPa)	Elongation
Test result	65.8	129.5	52	24.2%
Standard value [21]	68.9	124	55.2	25%
Relative error	4.5%	4.4%	1.9%	3.2%

**Table 4:** The parameters of biaxial tension under different speeds.

No.	Tension Speed	Specimen No.	Data No.	X Anti-Tensile Limit	Y Anti-Tensile Limit
1	20 μm/min	1#	SZ01	381.80 MPa	404.60 MPa
2	40 μm/min	2#	SZ02	388.31 MPa	406.31 MPa
3	60 μm/min	3#	SZ03	394.30 MPa	415.84 MPa
4	80 μm/min	4#	SZ04	402.39 MPa	429.02 MPa
5	100 μm/min	5#	SZ05	402.53 MPa	430.06 MPa
