# Correction: Li, H. et al. Research on Design and Simulation of Biaxial Tensile-Bending Complex Mechanical Performance Test Apparatus. *Micromachines*, 2017, *8*, 286

**DOI:** 10.3390/mi8100303

**Published:** 2017-10-12

**Authors:** Hailian Li, Hongwei Zhao, Chunyang Luo, Lijia Li, He Zhang

**Affiliations:** 1 School of Mechanical Science & Engineering, Jilin University, Changchun 130025, China; hlli12@mails.jlu.edu.cn (H.L.); llj15@mails.jlu.edu.cn (L.L.); hez15@mails.jlu.edu.cn (H.Z.); 2 School of Mechanical Science & Engineering, Beihua University, Jilin 132021, China; luochunyang2004@126.com

In the published paper [1], there is an error in Figure 8. The labels in Figure 8 was incorrect, it should be corrected as follows:

■—Characteristic point A, Elastic deformation;
▲—Characteristic point B, Elastic deformation;
●—Characteristic point C, Elastic deformation;
□—Characteristic point A, Plastic deformation;
△—Characteristic point B, Plastic deformation;
○—Characteristic point C, Plastic deformation;

The correct figure should read as follows:

**Figure 8:**
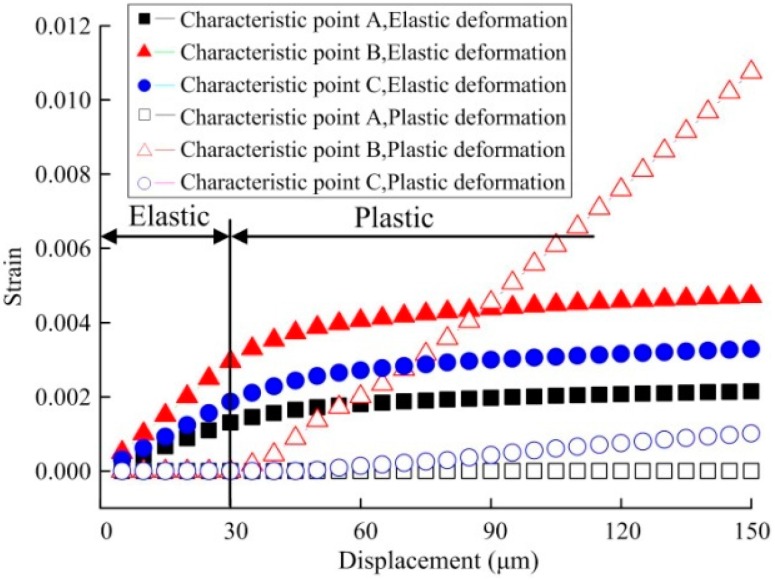
Displacement-dependent variation for strain of the specimen under biaxial tensile load.

The authors apologize for any inconvenience caused by the error. The manuscript will be updated online and the previous version will remain available on the article webpage.
